# Group B streptococcus (GBS) is an important pathogen in human disease- but what about in cystic fibrosis?

**DOI:** 10.1186/s12879-017-2729-6

**Published:** 2017-10-02

**Authors:** Kate Skolnik, Austin Nguyen, Christina S. Thornton, Barbara Waddell, Tyler Williamson, Harvey R. Rabin, Michael D. Parkins

**Affiliations:** 1 Department of Medicine, University of Calgary, 7007 14th Street SW, Calgary, AB T2V 1P9 Canada; 2 Department of Microbiology, Immunology and Infectious Diseases, University of Calgary, 3330 Hospital Drive NW, Calgary, AB T2N 4N1 Canada; 3 Department of Community Health Sciences, University of Calgary, Third Floor TRW Building, 3280 Hospital Drive NW, Calgary, AB T2N 4Z6 Canada

**Keywords:** Cystic fibrosis, *Streptococcus agalactiae*, Group B streptococci, Pulmonary exacerbation, Pneumonia

## Abstract

**Background:**

Group B Streptococcus (GBS) is a common commensal capable of causing severe invasive infections. Most GBS infections occur in neonates (often as pneumonia). GBS can also cause infection in adults with diabetes and other immunological impairments but rarely leads to pneumonia in adults. GBS has occasionally been found in the sputum of Cystic Fibrosis (CF) patients, an inherited condition known for progressive lung disease. However, the epidemiology and clinical significance of GBS in CF are not understood.

**Methods:**

We retrospectively reviewed a large single-centre adult CF population with an associated comprehensive, prospectively collected bacterial biobank beginning in 1978. We identified all individuals with GBS isolated from their sputum on at least one occasion. The primary outcome was risk of pulmonary exacerbation (PEx) at the time of the first GBS isolate compared to the preceding visit. Secondary outcomes included determining: prevalence of GBS infection in a CF population, whether GBS infections where transient or persistent, whether GBS strains were shared among patients, change in % predicted FEV_1_ at the time of GBS isolate compared to the preceding visit, PEx frequency after the first GBS isolate, change in % predicted FEV_1_ after the first GBS isolate, and complications of GBS infection.

**Results:**

GBS was uncommon, infecting 3.5% (11/318) adults within our cohort. Only three individuals developed persistent GBS infection, all lasting > 12 months. There were no shared GBS strains among patients. PEx risk was not increased at initial GBS isolation (RR 5.0, CI 0.69–36.1, p=0.10). In the two years preceding initial GBS isolation compared to the two following years, there was no difference in PEx frequency (median 2, range 0–4 vs 1, range 0 to 5, respectively, p=0.42) or lung function decline, as measured by % predicted FEV_1_, (median −1.0%, range −19 to 7% vs median −6.0%, range −18 to 22%, p=0.86). There were no invasive GBS infections.

**Conclusion:**

In adults with CF, GBS is uncommon and is generally a transient colonizer of the lower airways. Despite the presence of structural lung disease and impaired innate immunity in CF, incident GBS infection did not increase PEx risk, PEx frequency, rate of lung function decline, or other adverse clinical outcomes.

**Electronic supplementary material:**

The online version of this article (10.1186/s12879-017-2729-6) contains supplementary material, which is available to authorized users.

## Background

Cystic Fibrosis (CF) is an inherited autosomal recessive condition that can affect multiple organs but is best recognized for chronic and progressive lung disease [1]. Dysfunction of the CFTR (cystic fibrosis transmembrane conductance regulator) protein channel, found primarily on epithelial cell surfaces, leads to abnormal chloride ion secretion [1]. This results in viscous mucus leading to ductal and luminal plugging in respiratory, gastrointestinal, and reproductive organs [1]. In the lungs, thick secretions impair mucociliary clearance, permitting bacterial colonization, recurrent infections, chronic inflammation, and culminating in structural lung damage [2].

The microbiome of CF lungs is diverse and can change over time [3]. While some microbes are transient, others, such as *Pseudomonas aeruginosa* and *Staphylococcus aureus* may become persistent colonizing organisms in the lungs [3]. Some bacteria more commonly seen in CF, such as *P. aeruginosa*, *Burkholderia cenocepacia*, and methicillin resistant *S. aureus* (MRSA), are known to have adverse clinical outcomes in CF [4–6]. However, the implications of other, “rare” pathogens are unclear [7]. Whereas certain “rare” CF organisms, notable for their obscurity in other areas of medicine, have received extensive study (ie Inquilnus [8], Pandoraea [9], Ralstonia [10]) large deficits exist with those more mundane organisms such as the Streptococci. Non-classical organisms are not typically tracked or reported in large national registries of CF pathogens [11–14]. Consequently, the natural history and clinical impact of many non-classical species are not understood in CF. Recent data from our group demonstrated that Group A Streptococci (GAS), while uncommon in CF, may contribute to PEx in individuals with CF when present in high abundance [15]. In contrast to GAS, the role of another hemolytic Streptococci, *Streptococcus agalactiae* (Group B Streptococcus (GBS), has not been investigated in CF.

GBS is a beta-hemolytic, catalase negative facultative anaerobe [16]. GBS colonizes the oropharynx and/or genitourinary tract in 10 to 30% of humans, but is also capable of causing severe infections [16, 17]. The greatest burden of disease from GBS falls on neonates, with it being the most common cause of neonatal bacteremia, pneumonia, and meningitis worldwide [18]. GBS is also known to cause severe invasive infections in adults. GBS infection has been reported in 12 per 100,000 pregnancies typically as endometritis, urinary tract infections, and occasionally, maternal bacteremia [17, 19]. GBS can also cause significant morbidity in non-pregnant adults; while relatively rare in younger individuals, invasive GBS infection incidence is 24 per 1,000,000 in those over age 65 [19]. In non-pregnant adults, GBS infection generally presents as soft tissue infection (22%), osteomyelitis/septic arthritis (18%), pneumonia (11%), or bacteremia without identifiable focus (48%) [17]. Rarely, it may lead to endocarditis, meningitis, or necrotizing fasciitis [17]. In adults, risk factors for invasive GBS infection include older age, cirrhosis, diabetes mellitus, heart disease, stroke, neurogenic bladder, sacral ulcer, and malignancy [17, 20]. Most adults with invasive GBS have at least one risk factor [17, 20]. Interestingly, structural lung disease has not been specifically studied as a risk factor. From a pulmonary perspective, GBS is a frequent cause of neonatal pneumonia but an uncommon contributor to community acquired pneumonia and empyema in adults [17]. When GBS causes pulmonary infections, it is generally identified as part of polymicrobial pneumonia [21, 22].

GBS has only rarely been reported in CF [23] [24]. We sought to clarify the epidemiology and investigate its clinical significance of GBS in the adult CF population.

## Methods

### Population

In this single centre retrospective cohort study, individuals with documented CF [25] and at least one sputum culture positive for GBS were included. All adults with CF enrolled in our clinic since 1978 were screened for eligibility based on their sputum microbiology. This was achieved by evaluating the Calgary Adult CF Clinic Biobank (CACFB), a prospectively collected and inventoried repository of every bacterial isolate from every CF sputum sample collected from each clinical encounter since 1978 as outlined previously by Skolnik, et al. [15]. Individuals under age 18 were excluded. Respiratory specimen data was limited to sputum samples (throat/cough swab and bronchoalveolar lavage cultures were not available). Informed consent for study participation was obtained from patients upon enrollment in the clinic. The Conjoint Health Research Ethics Board (E-23087) granted ethical approval for the study.

### Clinical data collection

The study encompassed the two years before and two years after the initial GBS isolate, modeled on a prior study design [15]. Data collection included information regarding patient demographics, CF and non-CF comorbidities, medications at the time of initial GBS isolate, PEx occurrence and treatment, and spirometric data. Quantitative microbiology (reported as colony forming unit (CFU)/ml of sputum) was performed on each sample as is standard of care at our institution.

### Outcomes

The primary outcome was to determine whether the risk of pulmonary exacerbation (PEx) was increased around the time of first GBS isolate compared to the preceding visit. As routine visits are typically quarterly, the preceding visit was generally three months prior. Secondary outcomes included determining: (1) prevalence of GBS infection in a CF population, (2) what proportion of GBS infections became persistent, (3) whether GBS strains were shared among patients, (4) change in % predicted FEV_1_ at the time of first GBS isolate compared to the preceding visit, (5) PEx frequency in the two years following first GBS isolate compared to the two preceding years, (6) change in % predicted FEV_1_ in the two years following first GBS isolate compared to the two preceding years, and (7) complications of GBS infection.

A PEx was defined based on meeting Fuchs criteria [26]. A severe PEx was defined by the need for IV antibiotics and/or hospitalization. Our group previously determined that retrospectively diagnosed exacerbations generally correlate well with Fuchs Criteria at our centre [27]. Longitudinal outcomes of lung function and PEx frequency were compared in the two years preceding the subjects’ first GBS isolate to the two following years (using subjects as their own controls).

Unlike *P. aeruginosa*, [28], pulmonary infections with other organisms in CF lack criteria for chronicity that are well delineated and widely adopted. We defined persistent infection as three or more positive cultures and/or > = 50% GBS positive sputum cultures in one year, modeled on prior epidemiologic studies in CF [28, 29]. GBS positive cultures had to be separated by at least two weeks to be considered as separate events. GBS positive cultures did not have to be consecutive to be considered persistent.

### Bacterial strain typing

Genotyping was performed to characterize GBS strains and determine if any were shared among patients (which may be suggestive of transmission among patients). Bacterial strain typing was performed by pulse-field gel electrophoresis (PFGE) using previously published protocols [15, 30, 31]. Dendrograms were generated at 1.0% position tolerance using the unweighted pair-group method with arithmetic mean (UPGMA) and the Sørensen-Dice similarity coefficient. Strains with banding patterns ≥80% identical (≤3 band differences) were considered potentially related, conforming to the Tenover criteria [26].

### Antibiotic susceptibility testing

Antibiotic susceptibility testing was performed as per prior study protocols [15, 32]. Colonies from a 24-h incubation on Columbia Blood Agar (CBA) were suspended in 0.85% saline solution to a 0.5 McFarland standard and spread on the Mueller-Hinton Blood Agar (MHBA) plates. The following antimicrobial discs (from Oxoid, Nepean, Ontario) were used: penicillin G (P, 10 U), ceftriaxone (CRO, 30 μg), ceftazidime (CAZ, 30 μg), azithromycin (AZM, 15 μg), erythromycin (E, 15 μg), clindamycin (CDA, 2 μg), and levofloxacin (LEV, 15 μg). After a 24-h incubation, zones of clearance were measured and resistance was assigned in accordance to breakpoints as provided by the Clinical and Laboratory Standards Institute (CLSI) [33]. The “D-test” for inducible clindamycin resistance was performed.

### Statistical analysis

Descriptive statistics were used to summarize GBS prevalence in the CF population and the baseline characteristics of the included cohort. The relative risk (RR) of PEx at initial GBS isolation compared to the preceding visit (without GBS) was determined using McNemar’s test. Chi squared testing was applied to determine if any of the following variables influenced RR of PEx at initial GBS isolate: sex, age, chronic inhaled antibiotic use, chronic oral antibiotic use, inhaled steroid (ICS) use, long acting bronchodilator (LABA) use, concurrent *P. aeruginosa,* GBS abundance relative to other microbes, or GBS density as measured by colony forming units (CFU). Age was treated as a dichotomous variable; the median age was used to divide the group into “younger” and “older” individuals.

As the sample was small and the data was not normally distributed, signed rank analyses were applied to compare % predicted FEV_1_ at initial GBS isolate compared to the preceding visit as well as lung function decline and PEx frequency before and after the initial GBS isolate. Lung function decline was calculated by finding the absolute difference in % predicted FEV_1_ at first GBS isolate and two years prior (or a date closest to this) and dividing by the number of years between the two time points to get a “preGBS slope for lung function over time” for individual subjects. This was replicated to obtain a “postGBS slope for lung function over time”. Signrank analysis was then applied to compare “preGBS slope for lung function over time” to “postGBS slope for lung function over time” for individual subjects. All statistical analyses were performed using STATA/IC 13.1 software (Stata- Corp, TX, USA).

## Results

### GBS prevalence and cohort characteristics

Thirty GBS isolates were identified within the biobank from 11 individuals (Table 1). Additional details regarding patient clinical features and lung function are available in Additional files 1 and 2. As 318 CF adults were followed by our centre between 1978 and 2013, this represented a GBS prevalence of 3.5%. There were a comparable number of men (6; 55%) and women (5; 45%) with GBS in their sputum. The median age at initial GBS isolation was 27 years (range 20 to 77). The majority of individuals with GBS were nonsmokers (91%), most (71%) were pancreatic insufficient and 27% (3/11) had CF-related diabetes (CFRD). Pertinent chronic medication used by subjects at the time of first GBS isolate were chronic inhaled tobramycin in 50% (*n* = 5), chronic non-tobramycin antibiotics in 30% (*n* = 3; two AZM and one minocycline), LABA in 50% (*n* = 5), and LABA/ICS in 10% (*n* = 1). None of the patients were on ICS alone. Medication information was not available for one subject.

**Table 1:** *S.agalactiae* Sputum Isolates and Exacerbation Status in Adult Cystic Fibrosis Patients

Isolate	Patient	Date of Isolation	CFU^a^	GBS Most Abundant	Reduced FEV_1_^b^	PEx^c^ *	ABx Required	Co-Infections^d^
1	1	1990	10^4^	N	N	Y	Y	HI
2	2	2005	10^4^	N	N	N	–	PA
3	3	1994	10^6^	N	Y	Y*	Y	MSSA, SM
4	4	2013	10^6^	Equal	N	N	–	PA
5	5	2012	10^6^	N	N	Y	Y	PA
6	5	−2012	10^4^	N	Y	N	–	PA
7	5	−2012	10^5^	N	N	N	–	PA
8	5	2012	10^6^	Equal	N	N	–	PA
9	5	2013	N/A	Equal	Y	N	–	PA
10	5	2013	10^5^	N	N	N	N	PA
11	5	2013	10^6^	N	N	Y	Y	PA
12	5	2013	10^7^	Y	Y	Y*	Y	PA
13	6	1992	10^5^	N	N	N	–	PA, MSSA
14	7	1986	10^4^	N	N/A	Y*	Y	PA
15	7	1986	N/A	Y	N	N	–	–
16	7	1986	N/A	Y	N	N	–	–
17	7	1987	10^6^	N	N	N	–	HI
18	7	1987	10^5^	N	Y	N	–	O
19	7	1987	10^5^	N/A	N	N	–	N/A
20	7	1987	10^4^	N	N	N	–	–
21	8	2011	10^5^	Equal	N	N	–	MSSA
22	9	2013	10^6^	Y	N	N	–	–
23	10	2013	10^6^	Equal	N	N	–	MSSA
24	11	1990	10^5^	N	N	Y	Y	PA
25	11	1991	10^6^	Y	N	N	–	PA
26	11	1992	10^6^	Equal	N	N	–	PA
27	11	1992	10^4^	Equal	N	Y	Y	PA
28	11	1993	10^8^	Y	N	Y	Y	PA
29	11	1993	10^7^	N	N	N	–	PA, O
30	11	1993	10^7^	Equal	N	N	–	PA, O

^a^CFU = colony forming units

^b^Reduced FEV1 = reduction in reduction in FEV_1_ by >10% and/or >200cm^3^ at time of isolate compared to baseline FEV_1_

^c^PEx = pulmonary exacerbation as defined by Fuch’s Criteria and/or need for antibiotics

^*^Severe PEx = pulmonary exacerbation requiring intravenous antibiotics and/or hospitalization

^d^HI: *Haemophilus influenza,* PA: *Pseudomonas aeruginosa,* MSSA: Methicillin sensitive *S. aureus, SM = Stenotrophomonas maltophilia, S.marcecens = Serratia marcecens,* Bc: *Burkholderia cenocepacia*, CA: *Candida albicans, O: other* N/A = Not available N = No Y = Yes

Persistent (but not necessarily consecutive) GBS isolation occurred in three individuals, with two eventually clearing spontaneously (after 20 months), and the third clearing after 36 months, the latter following multiple antibiotic therapies for PEx. One patient had 9 positive cultures on separate occasions spanning 20 months.

### GBS genotype and antibiotic susceptibility

**Fig. 1:**
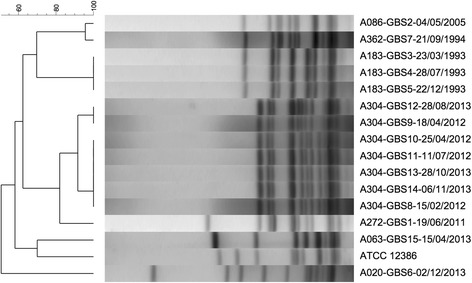
SmaI restriction digest pulse field gel electrophoresis of *S. agalactiae* recovered from CF sputum. A = patient number, date indicated as day/month/year. ATC 12386 indicates a control

PFGE was performed on 15 isolates derived from 7 individuals, demonstrating the same strain persisted in individual patients despite gaps in positive cultures (Fig. 1). However, there was no evidence of shared lineages. When screened prospectively all isolates demonstrated susceptibility to penicillin. All recoverable strains were susceptible to penicillin, ceftriaxone, and levofloxacin; approximately half (8/15) were macrolide resistant. Two individuals were on chronic azithromycin for PEx prevention; one had a macrolide sensitive GBS strain while the other was a resistant strain. All macrolide resistant isolates (8/15) had inducible clindamycin resistance.

### GBS infection and short term clinical outcomes

At the time of index GBS isolation, 45% (5/11) of individuals experienced a PEx and under half were severe. Of all occasions when GBS was isolated, 30% (9/30) associated with PEx and one third were severe. The relative risk (RR) of PEx at initial GBS isolate was higher than the preceding visit but not statistically significant (RR 5.0, CI 0.69–36.1, *p* = 0.10). RR of PEx at first GBS isolation was not affected by patient age, sex, chronic nebulized antibiotic use, chronic oral antibiotic use, LABA use, *P. aeruginosa* co-infection, if GBS was the numerically predominant organism, or if GBS was present at a particular CFU (data not shown). Although bacteria other than *P. aeruginosa* were co-isolated with GBS, associations between GBS and these organisms were not examined as the numbers were too small to provide a meaningful analysis. With initial GBS isolation, subjects did not experience a significant change in % predicted FEV_1_ compared to their preceding clinic visit (generally 3 months prior) (*p* = 0.50).

### GBS infection and long-term clinical outcomes

PEx frequency in the two years preceding initial GBS isolate was similar to the two following years (median 2, range 0 to 4 vs 1, range 0 to 5, respectively *p* = 0.42). Similarly, the frequency of severe PEx did not meaningfully change (median 0, range 0 to 1 vs 0, range 0 to 2, *p* = 0.95). Longitudinal lung function decline, as measured by % predicted FEV_1_, did not change significantly in the two years following initial GBS isolation (median − 1.0%, range − 19 to 7%) compared with the two prior years (median − 6.0%, range − 18 to 22%, *p* = 0.86). No patients developed complications such as pneumonia, bacteremia or empyema. No patient received antibiotics merely because of GBS isolation.

## Discussion

We aimed to clarify the epidemiology and clinical implications of GBS in an adult CF population. In our study, GBS was not commonly observed– infecting only 3.5% (11/318) patients over four decades of surveillance. GBS prevalence was slightly lower than that for GAS (4.7% observed in the same cohort) [15]. The literature on beta- hemolytic streptococci prevalence in CF is limited [15, 23, 24, 34]. Only two previous studies specifically reported prevalence of GBS pulmonary infection in CF [23, 24]. Doern et al. found a GBS prevalence of 0.7% (2/258) in an investigation of consecutive CF sputum samples with a comparable prevalence of other beta-hemolytic streptococci in the same cohort [24]. However, the GBS genotype and phenotype, patient characteristics, and clinical outcomes were not explored [23]. In contrast, Eickel et al. found a GBS prevalence of 16% (30/185) in German CF patients over a four-year period [23]. Our reported GBS prevalence is closer to that reported by Doern et al. [24] but lower than that of the German study [23]. The reason for the discrepancy is unclear; laboratory protocols for GBS isolation were comparable between these studies and ours. The younger age of the German CF patients compared to our cohort could be a potential explanation for the GBS prevalence differences. It is also possible that GBS oropharyngeal colonization patterns in Canadian versus German adults could be different in the general populations, however, this data is not readily available. Interestingly, the prevalence of GBS maternal colonization and incidence of neonatal infections in Germany is comparable to international estimates [35–38]. Unfortunately, the prevalence of many non-classical CF pathogens including beta hemolytic streptococci is not reported in national CF registries as yet [11–14].

In terms of characteristics of patients with GBS, we did not find GBS had a predilection for sex in CF patients, in keeping with prior literature [23]. The median age of our GBS cohort at initial isolate was slightly higher than in Eickel’s study, however we did not compare clinical characteristics of GBS positive patients to GBS negative patients. Interestingly, 27% (3/11) of our patients with GBS infection had CFRD at the time of isolation in contrast to none in Eickel’s study [23], which may have to do with the older age of our cohort. While diabetes is a risk factor for GBS infection in the general population [17, 20], it did not seem to account for the difference in GBS prevalence between our CF cohort and that of Eickel et al. [23]. Although our study was not designed to look at risk factors for GBS pulmonary infection in CF, it would be prudent for future studies to examine this.

With respect to GBS typing, we found that in those subjects with repeated GBS isolation, the same strain persisted within patients. However, strains were not shared between patients, indicating that patient-to-patient transmission had not occurred. Our GBS strains had a similar antibiotic susceptibility profile to those of the German CF population [23, 39]. Importantly, higher rates of antibiotic resistance were observed in CF patients relative to rates reported in the general population, demonstrating the impact of frequent antibiotic exposures in CF on commensal flora [40].

An additional similarity between our study and that of Eickel et al. was the presence of other bacterial species co-colonizing along with GBS [23]. Interestingly, our GBS cohort had a higher percentage of *P. aeruginosa* as a co-pathogen (90%, 10/11) compared to the German CF cohort with GBS (25%, 4/16) [23], raising the possibility that GBS may be more likely to cause pulmonary infection in individuals without chronic *P. aeruginosa* infection [27].

While persistent GBS infection occurred in 27% (3/11), all eventually cleared (generally without antibiotics). In most, GBS was only observed transiently despite a long follow-up period, compared with classic CF bacteria such as *P. aeruginosa,* which tend to cause chronic infection. In contrast, Eickel et al. found 44% of those with GBS (7/16) had persistent pulmonary infection lasting a median of 2 years (maximum 3 years) [23]. Of note, unlike *P. aeruginosa,* the natural history of other CF bacterial infections is not as predictable, therefore the “chronic infection” criteria used for *P. aeruginosa* may not be directly transferable to other CF pulmonary infections [28]. Furthermore, “chronic” may not necessarily be the best term to describe prolonged non-*P. aeruginosa* infections as some may be intermittently or continuously be present for long periods and then disappear. This highlights the importance of long-term studies to understand the natural history of airways infections in CF, rather than merely applying definitions of “chronic infection” derived from *P. aeruginosa* [28].

Our study is unique as it is the first to systematically investigate short and long-term clinical outcomes in adults with CF and GBS in their respiratory secretions. Although GBS is a pathogen known for its potential for severe invasive disease and CF is characterized by structurally damaged lungs with impaired innate immunity, the risk of pulmonary complications (including PEx occurrence at first GBS isolate, lung function decline at first GBS isolate, PEx frequency and lung function over time) as well as invasive GBS infections were not elevated in this small single centre study. Eickel et al. noted that half (12/24) of their clinic visits where CF subjects were GBS positive were associated with PEx [23]. Although this was not a pre-specified outcome in our analysis, on review of our individual GBS isolates, 30% (9/30) were associated with concurrent PEx. However, most (6/9) of these GBS isolates occurred in two chronically colonized individuals, one of whom had end-stage lung disease. Furthermore, there are other factors that could influence exacerbations such as compliance, asthma, viral infections and other bacterial pathogens. Overall, our results are reassuring as pulmonary GBS infection did not lead to negative short or long-term outcomes in adults with CF.

Our study has several limitations. Although we reviewed 318 CF patients for GBS, this is still a relatively small population to investigate an uncommon organism in a rare disease. However, this is offset by the very large number of patient-years of observation afforded through a four decade study. Nevertheless, a sample of 11 patients is small and less likely to detect small detrimental effects secondary to GBS, even if they are truly present. The retrospective design is also a limitation as clinical data may not be entirely complete; not all patients had exactly 2 years of data preceding and following the first GBS isolate, which could influence the accuracy of long term outcome estimates but did not invalidate the study. Laboratory limitations included the inability to grow all of the isolates for genotyping. Since only GBS isolates from 7/11 subjects could be genotyped (and only 2 of 3 subjects with persistent GBS were captured), it is possible that these other subjects had shared GBS strains amongst themselves and/or the same subject harbored different GBS strains. However this limitation did not affect clinical outcomes analyses. Our work was also limited by the fact that GBS identification is dependent on identifying beta-hemolytic Streptococci in sputum samples. However, as beta hemolysis is dependent on presence of functional *cyl* gene cluster, (present in 97% of strains), it is unlikely that non-hemolytic GBS failed to be identified in some patients [36, 39]. Lastly, as the study excluded pediatric patients, we do not know if GBS has different epidemiology and outcomes in this CF population. Despite these limitations, our investigation has shed light on the epidemiology and clinical significance of GBS in CF lungs.

## Conclusion

In adults with CF, GBS is a transient colonizer in most instances. The risk of PEx at initial GBS isolation was not significantly different compared to the preceding clinic visit. Thus, there was no definitive evidence of adverse short term effects from GBS on CF subjects. There were no adverse effects on longitudinal lung function, PEx frequency or invasive disease. No strains were shared amongst patients suggesting endogenous acquisition as opposed to patient-patient spread. Studies with larger samples (and ideally, controls) are needed to confirm the negligible impact of GBS on the CF population as our sample may have been underpowered to detect a small or modest clinical impact. However, based on the current evidence, there is some reassurance that GBS colonization alone does not necessitate antibiotic treatment, even in this higher risk population.

## Additional files

Additional file 1: Clinical and microbiologic characteristics of individuals with CF at the time of first GBS positive sputum culture. (XLSX 39 kb)

Additional file 2: Longitudinal spirometric data for individuals with CF and GBS positive sputum culture. (XLSX 52 kb)

## Abbreviations

Bc: *Burkholderia cenocepacia*
CA: *Candida albicans*
CFU: colony forming units
GAS: group A streptococcus
GBS: group B streptococcus
HI: *Haemophilus influenza*
MSSA: Methicillin sensitive *S. aureus*
O: other
PA: *Pseudomonas aeruginosa*
PEx: pulmonary exacerbation
PFGE: pulse field gel electrophoresis
R: resistant
S: susceptible
*S.marcecens*: *Serratia marcecen,s*
SM: *Stenotrophomonas maltophilia*
